# *Hesperinus ninae* Papp & Krivosheina (Diptera: Hesperinidae) from Georgia: the second record of this peculiar species

**DOI:** 10.3897/BDJ.1.e1023

**Published:** 2013-12-03

**Authors:** Olavi Kurina

**Affiliations:** † Institute of Agricultural and Environmental Sciences, Tartu, Estonia

**Keywords:** Diptera, Hesperinidae, *Hesperinus ninae*, distribution, Georgia, Caucasus

## Abstract

A second record of *Hesperinus ninae* Papp & Krivosheina, 2010 is given on the basis of material collected by sweep net from the northern slope of the Saguramo range north of Tbilisi, Georgia. The habitus and male terminalia are illustrated and the systematics briefly discussed.

## Introduction

The Hesperinidae is a small relict family of nematocerous Diptera that includes only eight extant species in a single genus – *Hesperinus* Walker (Krivosheina 1997, Papp 2010). Pape (Pape 2009) described the family as almost endemic to the Palaearctic region. In addition to six Palaearctic species, just one Nearctic and one Neotropical species have been discovered (Papp 2010). Three species have been described from Eocene Baltic amber (Skartveit 2009) and two specimens in poor condition are known from Oligocene sediments in France (Nel and Skartveit 2012). According to a recent overview by Papp (Papp 2010), three species occur in the Western Palaearctic, viz. *Hesperinus imbecillus* (Loew 1858) in Austria, Italy, Slovenia, Croatia, Serbia, Albania, Romania, Bulgaria, *Hesperinus graecus* Papp 2010 in Greece, and *Hesperinus ninae* Papp and Krivosheina 2010 in Russian North Caucasus. Members of the genus *Hesperinus* are medium-sized, dark coloured, with long antennae, legs and abdomen. The wings are well developed in the males but are shortened in the females of some species including *Hesperinus ninae*, thus making it somewhat peculiar among nematocerous flies (Papp 2010). Very little is known of their biology except that the adults are scarce and collected mostly near streams in woodlands (Papp 2010). The larvae and pupae of an eastern Palaearctic species were found in decaying wood of deciduous trees (Krivosheina and Mamaev 1967).

## Materials and methods

**Figure 1.**
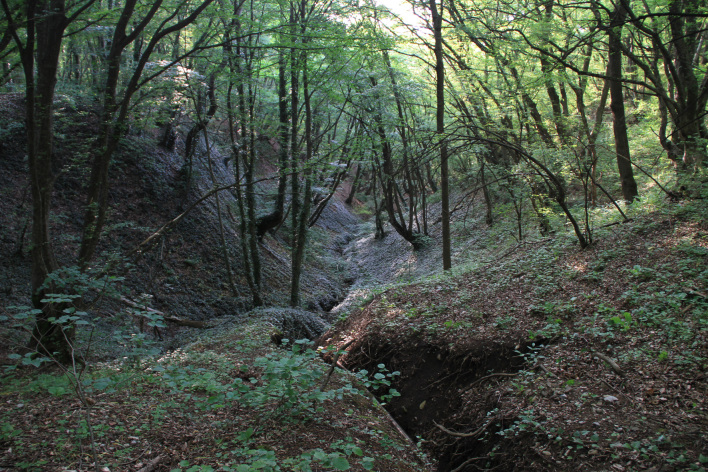
Collecting locality on the northern slope of the Saguramo range north of Tbilisi. Photo by O. Kurina.

The material reported here was collected during a recent expedition to Georgia in 2013. The locality lies on the northern slope of the Saguramo range (north of Tbilisi), covered by deciduous forests consisting of hornbeam, oak and maple. The sweep netting took place in vegetation along a narrow ravine with a steeply gullied bottom (Fig. 1). The collected specimens were either micro pinned or preserved in ethyl alcohol. Preparations of the male terminalia, as well as the illustrations given here (Figs 2, 3), were prepared using the methods and equipment described by Kurina and Oliveira 2013. All the material is deposited in the insect collection of IZBE—Institute of Agricultural and Environmental Sciences, Estonian University of Life Sciences (former Institute of Zoology and Botany), Tartu, Estonia.

## Taxon treatments

### Hesperinus ninae

Papp & Krivosheina, 2010

#### Materials

- **Type status:** Other material. **Occurrence:** recordedBy: Olavi Kurina; individualCount: 12; sex: male; **Location:** country: Georgia; verbatimLocality: Saguramo north of Tbilisi; verbatimElevation: 915; verbatimLatitude: 41°53’04,3’’N; verbatimLongitude: 44°46’46,5’’E; **Event:** samplingProtocol: sweeping; eventDate: 15 May 2013; **Record Level:** institutionCode: EMY; collectionCode: IZBE

#### Taxon discussion

*Hesperinus ninae* (Figs 2, 3) was described from two male specimens collected in the 1960s from Krasnaya Polyana (Krasnodar Kray in Russian North Caucasus), but according to Papp & Krivosheina (Papp and Krivosheina 2010) other material from the same collecting series had already been included in an overview by Mohrig et al. (Mohrig et al. 1975: as *Hesperinus imbecillus*).

Using the key by Papp (Papp 2010), the studied specimens run well to *Hesperinus ninae* because of the elongated terminal flagellomere (Fig. 2c), wing length about 6 mm (Fig. 2b), considerably shorter and broader first flagellomere with specific setation (Fig. 2d), and gonostylus mediodorsally with a projecting lobe (Fig. 3c). Like the European *Hesperinus imbecillus*, the females of *Hesperinus ninae* are flightless. In spite, that there were no females included to the original description (Papp and Krivosheina 2010), four of them from the same collecting series were studied by Mohrig et al. (Mohrig et al. 1975, as *Hesperinus imbecillus*). They figured a female with reduced wings (Mohrig et al. 1975: fig. 1) and did not described any differences between Northern Caucasian and Central European material (see also discussion by Papp and Krivosheina 2010). The flightlessness has obviously been an adaptive response to unfavourable climatic conditions and has set further limits to dispersal. However, the current record is at quite a remote distance from the type locality, indicating that the species probably has a wide distribution in suitable habitats in the Caucasus.

## Supplementary Material

XML Treatment for Hesperinus ninae

## Figures and Tables

**Figure 2a.**
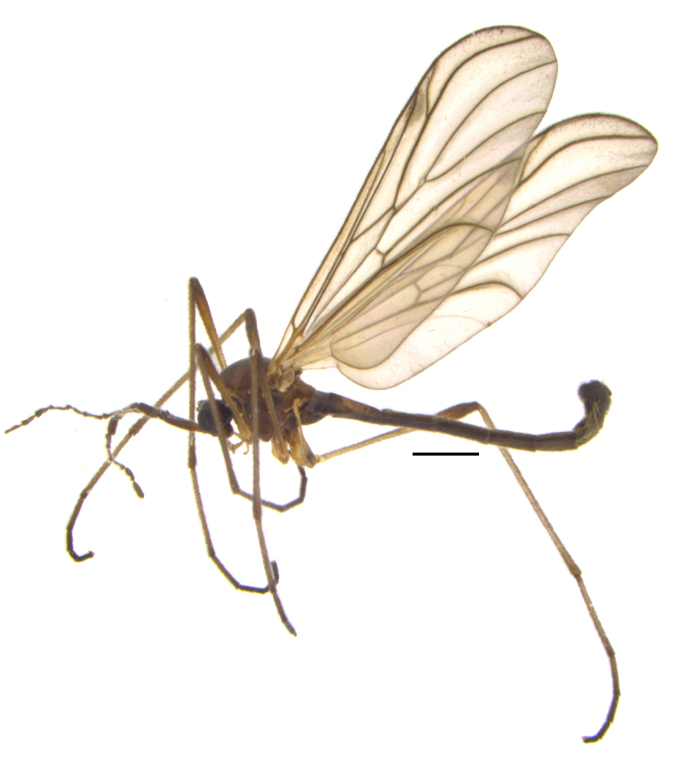
Male habitus, scale bar 1 mm.

**Figure 2b.**
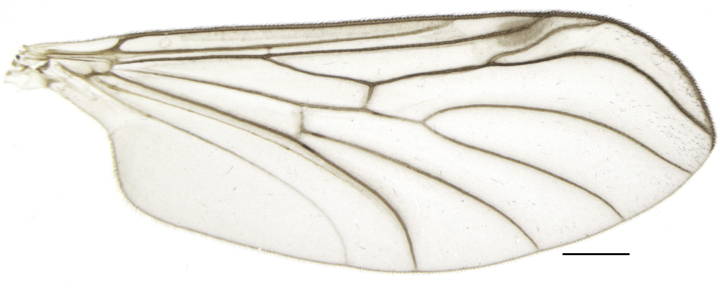
Wing, scale bar 1 mm.

**Figure 2c.**
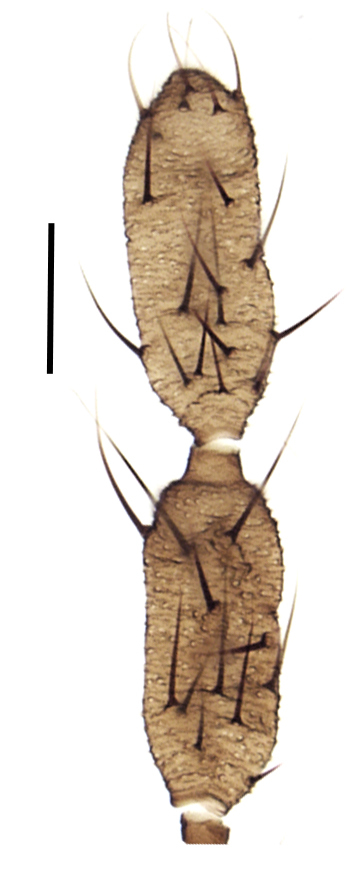
Penultimate and terminal flagellomeres, scale bar 0.1 mm.

**Figure 2d.**
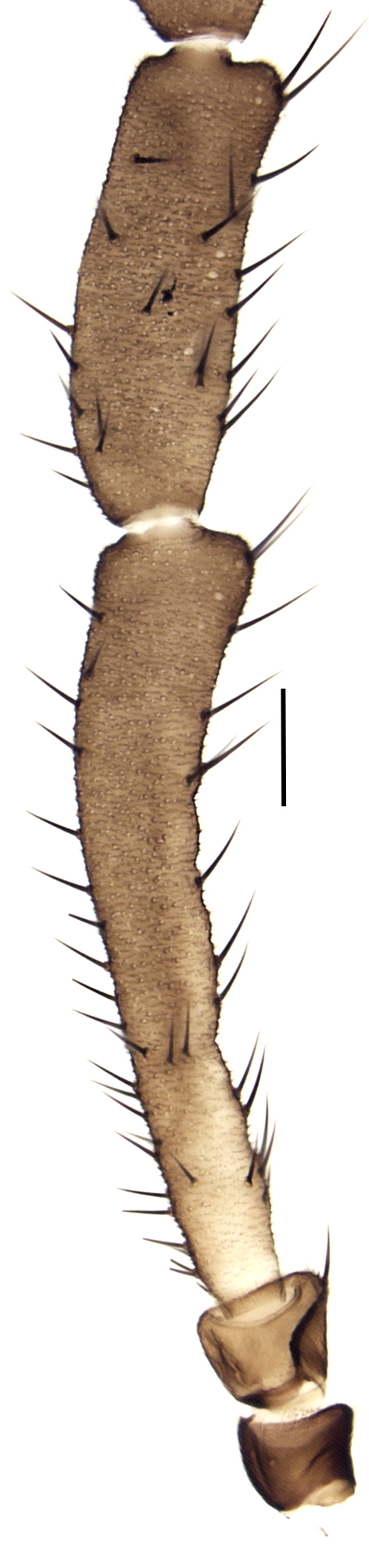
Scape, pedicel, first and second flagellomeres, scale bar 0.1 mm.

**Figure 3a.**
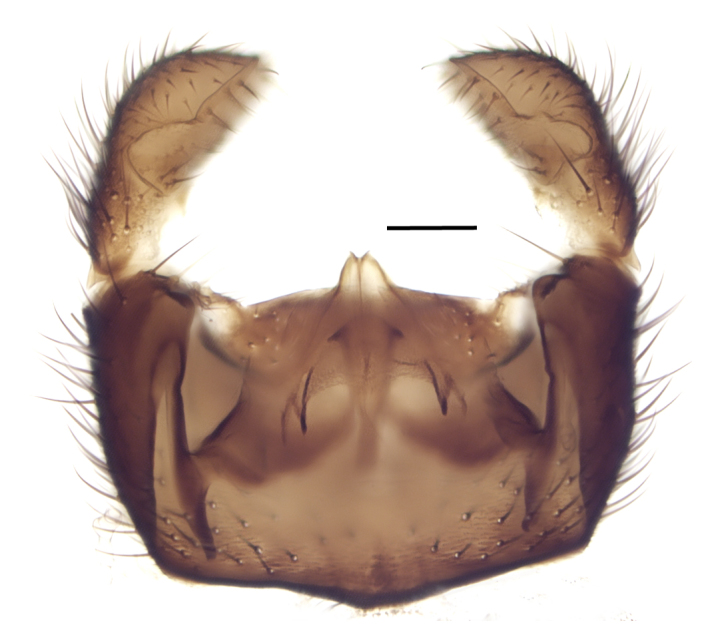
Dorsal view, tergite 8 detached, scale bar 0.1 mm.

**Figure 3b.**
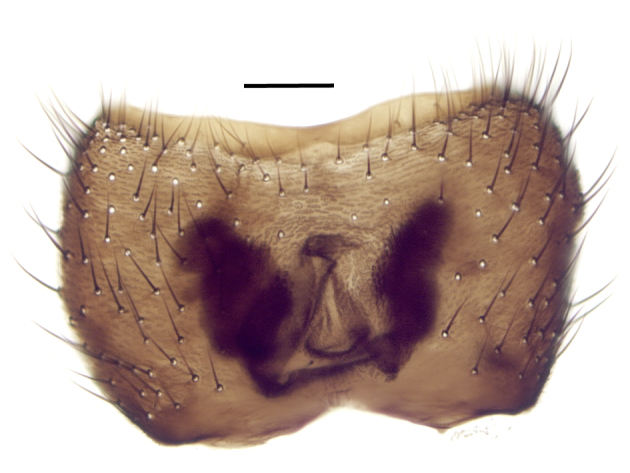
Dorsal view of tergite 8, scale bar 0.1 mm.

**Figure 3c.**
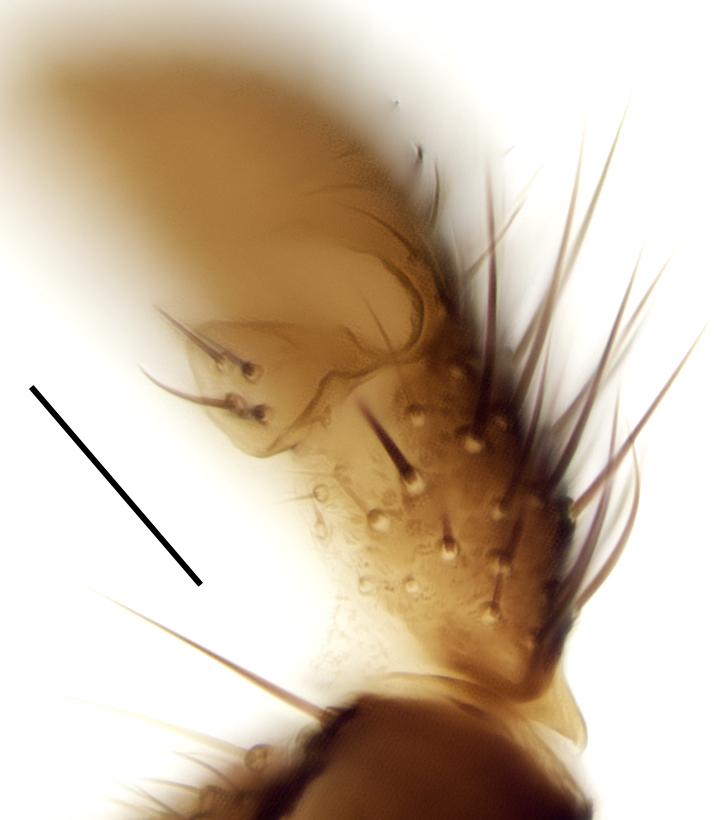
Closer view of gonostylus, scale bar 0.1 mm.
